# A Qualitative Exploration of Participants' Experiences in a Randomized Controlled Trial of Weight Reduction Through a Nurse-Led Intervention

**DOI:** 10.7759/cureus.69621

**Published:** 2024-09-17

**Authors:** Vembu Krishnasamy, Kumari Manjini Jayaram, J Venkatachalam

**Affiliations:** 1 Department of Community Health Nursing, Faculty of Nursing, College of Nursing, Jawaharlal Institute of Postgraduate Medical Education and Research, Puducherry, IND; 2 Department of Medical Surgical Nursing, College of Nursing, Jawaharlal Institute of Postgraduate Medical Education and Research, Puducherry, IND; 3 Department of Preventive and Social Medicine, Jawaharlal Institute of Postgraduate Medical Education and Research, Puducherry, IND

**Keywords:** in-depth interview, nurse-led intervention, participants' experiences, qualitative study, weight reduction

## Abstract

Background

Obesity presents a significant global health challenge, increasing the risk of various health complications. Nurse-led interventions offer a promising, patient-centered approach to weight reduction. However, qualitative research on participants' experiences in nurse-led weight reduction programs is limited.

Objective

The objective of this study was to explore the lived experiences of participants in a nurse-led weight reduction program over a period of 12 months.

Methods

Following a randomized controlled trial within a sequential mixed-methods study, in-depth interviews were conducted with 13 volunteers who successfully reduced their weight by at least 5% through a nurse-led intervention (NLI). The data were analyzed using interpretative phenomenological analysis, with careful attention to ensuring both data credibility and participant confidentiality.

Results

Participants reported positive health improvements over 12 months, including reductions in weight, BMI, waist circumference, blood pressure, and glucose levels. Four primary themes that surfaced were nutritional choices and habits, active lifestyle, support system and motivation, insights, and strategies from health journey participants. Participants adapted to healthier eating habits, incorporating protein-rich foods, millets, and seasonal fruits and vegetables. Walking and yoga were primary physical activities, with participants emphasizing their importance and strategies to overcome barriers. Family support, healthcare guidance, and work-life balance played crucial roles in participants' weight loss journeys.

Conclusion

The study highlights the effectiveness of NLIs in promoting sustainable weight loss and improving metabolic health. Insights from participants' experiences underscore the importance of dietary modifications, regular physical activity, and social support in successful weight management. Further research is needed to validate these findings and develop tailored interventions.

## Introduction

Obesity remains an escalating global health dilemma, with its prevalence surging in recent decades and posing a significant risk for various health complications, including cardiovascular diseases, diabetes, and specific cancers [1]. Consequently, weight reduction interventions have garnered substantial attention within public health strategies. It is now a priority concern to manage obesity, and primary care nurses may be significant players in this regard [2]. Nurses, occupying a unique position within healthcare settings, are well-positioned to deliver personalized care, education, and support to individuals on their weight loss journey.

Despite the growing interest in nurse-led weight management initiatives, there exists a paucity of qualitative research investigating participants' experiences with these interventions. This study seeks to address this gap by exploring the lived experiences of individuals engaged in a nurse-led weight reduction program, thereby offering invaluable insights into the effectiveness and acceptability of such intervention [3]. Adopting a qualitative methodology facilitates a nuanced understanding of the determinants influencing participants' adherence, their perceptions of the intervention's efficacy, and the challenges encountered during the weight loss process. Moreover, the findings from this study hold the potential to inform healthcare professionals, policymakers, and stakeholders about the merits of nurse-led approaches in weight management and their potential integration into routine clinical practice. This knowledge could catalyze the refinement of support strategies, enhance patient engagement, and address barriers to adherence, ultimately contributing to the advancement of patient-centered care in weight management.

Recently, a randomized controlled trial (RCT) was conducted to evaluate the efficacy of nurse-led weight-loss interventions, focusing on lifestyle modifications, particularly diet and exercise [4]. Participants in the NLI arm received personalized education on diet and physical activities, with individualized calorie requirement calculations and encouragement to maintain a minimum 200Kcal deficit. Additionally, self-monitoring equipment, including household weighing scales, nutritional cups, and pedometers, was provided. Yoga sessions centered on Surya Namaskar and nutrition exhibitions were conducted area-wise. Midline evaluations were performed at six months, with sessions repeated at nine months, culminating in an end-line evaluation at the 12-month mark. The data demonstrated significant weight loss in the NLI arm compared to the control group. The mean percentage weight difference between arms was 3.3 (95% CI 3.03, 3.50) at 6 months and 6.2 (95% CI 5.84, 6.54) at 12 months.

The objective of the present qualitative study was to uncover the general experiences of participants who achieved a 5% weight loss through the NLI over a period of 12 months.

## Materials and methods

Research team and ethical approval

The primary researcher (first author) is a nurse pursuing a doctorate in nursing with a focus on weight reduction among adults. The study was ethically approved by the Institute of Ethics Committee for Nursing Studies (IEC No. JIP/CON/IEC/2021, dated 20.10.2021) The trial has been registered prospectively with the Clinical Trials Registry India (CTRI) under the identifier CTRI/2021/12/038785 on 21st December 2021 [4]. The study report adhered to the Standards for Reporting Qualitative Research (SRQR) checklist for reporting qualitative research [5].

Overview of the RCT on weight reduction through the nurse-led intervention (NLI)

The RCT compared the NLI group, which received tailored diet and exercise interventions from the nurse researcher, to the general care group, which received standard care through urban primary health centers. The protocol for the presented trial is detailed elsewhere [4]. A total of 438 adults were recruited from urban areas in Puducherry. The inclusion criteria comprised adults aged between 18 and 50 years, residing in urban areas of Puducherry for at least a year, with a BMI of 25 kg/m² or more. Exclusion criteria encompassed individuals with limitations for physical activities, medication affecting weight loss, severe diseases like heart failure, liver failure, renal failure, etc., visual or hearing impairments, ongoing weight loss programs, bariatric surgery, pregnancy, and psychiatric disorders.

The NLI was developed based on recommendations from various specialists, including adult health nursing, endocrinology, general medicine, dietetics, physiotherapy, and medical social work. The intervention was structured through focus group discussions, considering factors aiding and hindering weight loss among overweight residents in the area. Pender's health promotion model served as the theoretical framework to promote adherence to diet and exercise [4]. Participants in the intervention arm received group-based educational sessions on healthy eating habits and physical activity, including weight reduction activities, during the initial two weeks. The teaching methods incorporated lectures, group discussions, and demonstrations of healthy food choices and Surya Namaskar exercises. BMI, ideal body weight, and calorie requirements were calculated using online health calculators [4]. A personalized meal plan was devised based on calorie requirements, biochemical characteristics, physical activity, and spending capacity on food. Participants were provided with self-monitoring tools, including Fitbit bands and nutritional measurement cups, and were encouraged to use mobile communication, participate in group activities, and receive counseling throughout the intervention.

Design

A qualitative approach utilizing interpretative phenomenological analysis (IPA) was employed to explore participants' experiences and perceptions [6]. IPA focuses on understanding how individuals make sense of their experiences and perceptions [7].

Recruitment and data collection procedure

The intervention was received by 219 participants in the NLI arm. In this qualitative study (IDI), the purposeful maximum variation sampling was used to recruit participants across various age groups, education levels, genders, and with varying weight loss success through the NLI [8]. Written informed consent was obtained from all participants, and face-to-face semi-structured interviews were conducted. The semi structured interview guide was pre-validated with experts. Interviews were recorded, transcribed, and analyzed until data saturation was achieved [9]. The data saturation was achieved with 13 interviews itself.

Data analysis

Data were analyzed using IPA to identify emergent themes and categories from the transcripts [10]. Measures were taken to ensure data credibility, dependability, and confidentiality. Peer verification, participant validation, and reflexivity were integrated to enhance the study's rigor and trustworthiness [11].

## Results

Participant demographics in the in-depth interview (IDI) study

Thirteen volunteers participated in this study. All participants were from urban areas of Puducherry and had received an NLI for weight reduction from February 2023 to January 2024. The participants described their overall experiences regarding their weight loss journey, including eating patterns, types of exercises, motivational factors, challenges faced during weight loss, how they overcame these challenges, and advice for those who have not yet taken steps to reduce weight.

The mean age of our sample population was 36.2 years with a standard deviation of 6.69. In terms of age distribution, the majority fell within the 29-30 age bracket with seven individuals, followed by the 40-50 age group with four individuals, and the 18-28 age group with two individuals. Religiously, all 13 individuals identified as Hindu. Regarding marital status, 11 individuals (84.62%) reported being married, while only two individuals (15.38%) indicated being unmarried. Educationally, the majority had completed primary education with seven individuals (53.85%), followed by graduates with five individuals (38.46%) and a single individual (7.69%) with secondary education. Occupation-wise, six individuals (46.15%) were housewives or unemployed, three individuals (23.08%) were skilled workers, and four (30.77%) held professional roles. In terms of family income, five individuals (38.46%) earned ≤25,000, six (46.15%) fell within the 25,001-50,000 bracket, and two (15.38%) had a family income exceeding 50,001 (Table 1).

**Table 1:** Demographic characteristics of in-depth interview (IDI) participants (n=13)

Sl. No.	Characteristics	Total no. of participants	Percentage	Cumulative percentage
1	Age in years (mean±SD)	36.2± 6.69		
	18-28	2	15.38	15.38
	29-30	7	53.85	69.23
	40-50	4	30.77	100.00
2	Sex			
	Male	2	15.38	15.38
	Female	11	84.62	100.00
3	Marital status			
	Unmarried	2	15.38	15.38
	Married	11	84.62	100.00
4	Education			
	Primary education	7	53.85	53.85
	Secondary education	1	7.69	61.54
	Graduates	5	38.46	100.00
5	Occupation			
	Housewife/ Unemployed	6	46.15	46.15
	Skilled	3	23.08	69.23
	Professionals	4	30.77	100.00
6	Family income (in rupees per month)			
	≤25000	5	38.46	38.46
	25001-50000	6	46.15	84.62
	>50001	2	15.38	100.00

Changes in physical activity and calorie intake among IDI participants during the NLI

The participants’ total physical activity measured in METS per week and daily calorie intake in kilocalories (Kcal), both at the beginning of the intervention (0 months) and after 12 months. Participants generally showed an increase in physical activity over the 12-month period. The median physical activity rose from 420 METS per week at 0 months to 1760 METS per week at 12 months (Q1-Q3: 320-640 at 0 months, 1480-2000 at 12 months), indicating a significant improvement in activity levels as a result of the intervention (Table 2).

**Table 2:** Changes measured in physical activity and calorie intake among in-depth interview (IDI) participants during the nurse-led intervention (n=13)

Participant ID	Total physical activity (METS/week)		Calorie intake (Kcal per day)	
	At 0 months	At 12 months	At 0 months	At 12 months
1	360	1480	2626	1843
2	320	1480	6413	2349
3	400	1480	3089	1895
4	800	1760	5094	2186
5	320	1360	4356 .	2326
6	420	1800	4323	2216
7	320	2040	3846	2457
8	200	960	3784	2346
9	5760	7680	5433	2524
10	2160	5520	5433	4632
11	480	2000	4399	2752
12	640	1120	4014	1854
13	640	1760	3457	2086
All participants median (Q1-Q3)	420 (320 -640 )	1760 (1480- 2000)	4323 (3784-5094)	2325.84 (2086 - 2457)

Calorie intake, on the other hand, demonstrated a decrease across most participants. The median daily calorie intake dropped from 4323 Kcal at the start of the intervention to 2325.84 Kcal at 12 months (Q1-Q3: 3784-5094 at 0 months, 2086-2457 at 12 months). This reduction suggests that participants were able to successfully lower their caloric consumption, contributing to their weight loss.

Overall, Table 2 highlights the positive impact of the NLI on both increasing physical activity and reducing calorie intake among the participants.

Changes in the clinical profile of IDI participants during the NLI

Over a 12-month period, participants exhibited positive health improvements in various metrics. Body weight reduced from an average of 87.9 kg to 80.6 kg, BMI decreased from 34.9 kg/m^2 to 32 kg/m^2, and waist circumference dropped from 101.4 cm to 95.7 cm. Systolic blood pressure declined from 124.2 mm Hg to 119.4 mm Hg, diastolic blood pressure reduced from 85.5 mm Hg to 78.8 mm Hg, and glucose levels decreased from 131.2 mg/dl to 115.5 mg/dl. Furthermore, lipid profiles showed improvements with total cholesterol decreasing from 220.7 mg/dl to 203.38 mg/dl, LDL cholesterol dropping from 153.8 mg/dl to 137.2 mg/dl, and triglycerides reducing from 151 mg/dl to 104.9 mg/dl. HDL cholesterol saw a slight increase from 43.8 mg/dl to 46.6 mg/dl, while VLDL cholesterol decreased from 30.2 mg/dl to 20.9 mg/dl. Additionally, TSH levels changed from 3.03 ulU/ml to 2.09 ulU/ml (Table 3)

**Table 3:** Changes in clinical characteristics of in-depth interview (IDI) participants during the nurse-led intervention (n=13) m: months

Participant ID	Body weight (Kg)		BMI (kg/m^2^ )		WC (cm)		Systolic BP (mm Hg)		Diastolic BP (mm Hg)		Glucose (mg/dl)		Total Cholesterol (mg/dl)		Triglycerides (mg/dl)		HDL (mg/dl)		LDL (mg/dl)		VLDL (mg/dl)		TSH (ulU/ml)	
	At 0 m	At 12 m	At 0 m	At 12 m	At 0 m	At 12 m	At 0 m	At 12 m	At 0 m	At 12 m	At 0 m	At 12 m	At 0 m	At 12 m	At 0 m	At 12 m	At 0 m	At 12 m	At 0 m	At 12 m	At 0 m	At 12 m	At 0 m	At 12 m
1	88	81.5	38.08	35.27	98.5	93.5	132	128	78	72	82	78	226	211	85	84	66	48	141	121	17	17	3.41	2.14
2	95	87.3	36.19	33.26	98	93.5	142	127	118	105	285	205	235	203	342	152	35	45	169	147	68	30	5.83	4.13
3	82.2	77.5	35.57	33.54	98	95	140	127	118	108	139	125	282	258	128	118	49	49	209	185	26	23	0.85	0.79
4	80	71.5	33.73	30.14	98	90	120	114	78	66	90	88	200	195	78	62	43	45	140	123	16	12	3.4	2.5
5	90	80.3	35.37	31.56	109	102	110	108	80	71	76	75	195	182	80	81	44	48	132	114	16	16	2.57	1.53
6	90	83	36.51	33.67	112	106	122	120	98	76	237	201	215	195	203	175	45	48	143	115	41	35	4.39	3.1
7	98	90.2	33.51	30.84	103	96.5	138	130	92	85	108	101	284	248	208	115	37	43	208	198	42	23	1.86	1.82
8	86	78	33.80	30.66	98	90	124	118	80	76	87	85	193	152	142	121	35	45	135	123	28	24	1.59	1.5
9	90.5	80.1	37.18	32.91	107	99.5	110	110	60	68	253	203	163	152	106	105	46	48	113	107	21	21	4.4	2.34
10	78	71.8	33.76	31.07	98	93	113	116	65	78	91	85	233	223	45	45	39	45	176	167	9	9	1.53	1.35
11	90	84.1	32.46	30.33	101	96	136	124	93	77	78	79	177	178	106	108	42	45	120	112	21	22	3.99	2.93
12	83	78	33.03	31.04	99.5	94	108	109	75	71	88	85	202	201	85	83	45	48	138	114	17	17	1.71	1.63
13	90.5	84.5	34.91	32.59	98.5	94.5	120	121	76	72	92	91	264	246	355	115	43	49	176	157	71	23	3.84	1.38
Mean±SD	87.9 ± 5.7	80.6± 5.4	34.9± 1.7	32 ± 1.6	101.4 ± 4.9	95.7 ± 4.5	124.2 ± 12.2	119.4 ± 7.6	85.5 ± 17.8	78.8 ± 13.2	131.2 ± 74.9	115.5 ± 51.5	220.7 ± 38.2	203.38 ± 33.9	151 ± 99.6	104.9 ± 35	43.8 ± 8	46.6 ± 2	153.8 ± 31	137.2 ± 30.5	30.2 ± 19.9	20.9 ± 7	3.03 ± 1.5	2.09 ± 0.9

Emerged themes

Weight management remains a significant concern for many adults, with numerous factors influencing their ability to maintain a healthy weight. NLIs have proven to be effective strategies in guiding individuals toward sustainable weight loss and improved health outcomes. This qualitative study delves into the experiences of adults who successfully lost at least 5% of their weight through NLIs, shedding light on the strategies, challenges, and motivations that shaped their health journeys. Four primary themes that surfaced were Nutritional Choices and Habits, Active Lifestyle, Support System and Motivation, Insights and Strategies from Health Journey Participants (Table 4).

**Table 4:** Themes and subthemes identified in adults achieving 5% weight loss through the nurse-led intervention

Theme	Subtheme	Categories	Codes
1. Nutritional Choices and Habits	1.1 Adapting to new eating habits and preferences	1.1.1 Diet composition and preferred choices	Inclusion of protein-rich foods and millets and drinking water
		1.1.2 Adoption of millets into diet	Consumption of millet-based dosa, and chapatti Consumption of millet with rice Consumption of millet-based porridge Preparation of millet-based snacks at home
		1.1.3 Integration of fruit & vegetables	Locally available green leafy vegetables. Incorporation of various vegetables into the daily diet. Regular consumption of seasonal fruits
	1.2 Food restrictions	1.2.1 Carbohydrate restrictions	Restriction of certain foods to manage health conditions Preference for jaggery over sugar in fruit juice. Reduction of high-carb cereals
		1.2.2 Oil restriction	Reduced oil; Used only for seasoning. Transition from frying to boiling or steaming
		1.2.3 Avoidance of unhealthy snacking and beverages	Cut out junk foods. Replacing tea and coffee with millet porridge Transition from consuming fast food to incorporating vegetables and millets. Avoiding excessive sweets
		1.2.4 Limiting non-vegetarian food intake	Limited consumption of non-vegetarian food
	1.3 Overcoming challenges related to dietary changes	1.3.1 Managing hunger	Lack of time. Opting for water, fruits, or fruit juice between meals
		1.3.2 Navigating dietary adaptation	Cooking healthy meals for the entire family. Positive outcomes from dietary and exercise changes
		1.3.3 Adapting to healthier choices	Initial resistance; Adaptation over time. Initial challenges in adapting to a vegetable-rich and low-rice diet. Overcoming initial challenges and staying motivated. Family-wide adoption of healthier dietary changes
2. Active Lifestyle	2.1 Incorporating exercise routines into daily life	2.1.1 A Routine of walking and Surya Namaskar	Engaging in walking regularly practice of Surya Namaskar
		2.1.2 Daily tasks, daily movement: continuous physical engagement	Integrating walking in job
		2.1.3 Flexible exercise timing based on availability	Evening walks, extended walking on holidays Daily walking, mostly in the evening
	2.2 Overcoming barriers with regular physical activity	2.2.1 Integrating yoga and Surya Namaskar into daily routines	Found yoga challenging initially
		2.2.2 Navigating physical constraints	Inability to practice due to physical limitations
		2.2.3 Adapting fitness routines	Successful adaptation to Surya Namaskar challenging due to flexibility issues
3. Support System and Motivation	3.1 Family support and work life balance	3.1.1 Family's shift towards valuing health and well-being	Spouse as the motivator Family transition
		3.1.2 Lifestyle changes without compromising family and work	Manage exercises without affecting family or work No impact on daily activities.
		3.1.3 Maintaining balance: health choices without sacrificing social connections	Daily walking; Involvement of family and friends. Walking for entertainment
		3.1.4 Family support	Family support; No disruption to family routines. Encouraging family members to adopt healthier habits
	3.2 Reflection and realization	3.2.1 Improved well-being through weight loss	Body feels lighter Feeling great about achievements, sense of happiness
		3.2.2 Healthy living essentials: exercise and nutrition's vital role	Realize the importance of exercise and proper nutrition Easy adaptation; Positive experience.
		3.2.3 Health benefits	Improvement in leg pain, breathing difficulties, and menstrual issues Potential savings on medical expenses through healthy lifestyle adoption Regular practice of yoga and Surya Namaskar, improvement in flexibility
4. Insights and Strategies from Participants	4.1 Strategies employed for achieving success	4.1.1 Guided choices: diet charts, daily routines.	Intent to continue lifestyle for weight maintenance. Emphasis on maintaining a daily routine for self-care controlled portions. Avoidance of fast food.
		4.1.2 Prioritizing health: Weight management, exercise, and proactive choices for well-being	Awareness of weight-related health risks. Promotion of self-health through daily workouts and avoiding health issues Encouragement for proactive steps. Avoidance of fast food
	4.2 Advice for weight loss and overall well-being	4.2.1 Seasonal choices: dietary recommendations for a healthier lifestyle	Eat seasonal fruits, avoid oily and packaged food Emphasizing balanced diet, vegetables, fruits, and regular exercise
		4.2.2 Small changes, big impact: beginning a sustainable weight loss journey	Start now, make small changes to diet and lifestyle Start today, even a 10-minute walk. Strict adherence to provided diet chart

Theme 1: Nutritional Choices and Habits

Nutritional Preferences and Practices delved into the dietary changes participants adopted with the support of NLIs. Participants described integrating protein-rich foods, millets, and a variety of fruits and vegetables, emphasizing the significance of mindful eating and making informed nutritional decisions.

Adapting to new eating habits and preferences: Participants highlighted their efforts to shift towards healthier eating habits. A significant emphasis was placed on the inclusion of protein-rich foods and millets in their diets.

"I've added protein-rich foods and millets to my diet." - IDI-5

"Had them raw, or sometimes had sprouted gram, chickpeas, etc." - IDI 6

Millets, in particular, were incorporated into various dishes such as dosa, chapatti, and porridge.

"I had Pearl millet Adai and wheat dosa. I also had chapati instead of poori." - IDI 6

"For lunch, I continued with rice, millets, and a variety of vegetables." - IDI-13

The increased consumption of water emerged as a common practice, with some participants starting their mornings with a glass of water.

“….and increased my water intake to about three to four litres per day."-IDI 12

"Firstly, I make it a point to wake up early and drink a full glass of water."- IDI 9

The integration of a diverse range of vegetables and regular seasonal fruits into daily diets was also emphasized. Homemade millet snacks were preferred over traditional store-bought options.

"I started incorporating drumstick leaves and vegetables into my meals." - IDI-1

"Additionally, I frequently eat fruits such as pomegranates, grapes, and watermelons." - IDI 10

Food restrictions: Participants detailed their dietary restrictions aimed at managing health conditions and adhering to specific guidelines. This included reducing carbohydrate intake, opting for natural sweeteners like jaggery over refined sugar, and limiting high-carb cereals.

"Initially, I reduced my idli intake from 6 to 4 and eventually down to 2. I also cut out tea, coffee, and milk from my morning routine." - IDI-1

A conscious effort was made to reduce oil consumption, with many participants choosing healthier cooking methods such as boiling or steaming. Unhealthy snacking and beverage consumption were curtailed in favor of vegetable and millet-based meals. Non-vegetarian food consumption was also restricted to varying degrees among participants.

Overcoming challenges related to dietary changes: Participants shared their experiences in adapting to new dietary habits.

"I adopted this dietary approach for my whole family as I didn’t have separate time to prepare different meals." - IDI-13”

Challenges included managing hunger, navigating dietary changes within family dynamics, and adjusting to healthier food choices. Despite these challenges, participants demonstrated determination and resilience. They highlighted the role of family support and shared positive outcomes from their dietary and exercise changes, reinforcing their motivation to continue their health journeys.

Theme 2: Active Lifestyle

In the theme of Active Lifestyle, the focus shifted to the exercise routines participants engaged in. Walking was identified as the primary form of physical activity, supplemented by practices such as Surya Namaskar, underscoring the essential role of physical activity in achieving and maintaining weight loss.

Incorporating exercise routines into daily life: Physical activity was a key focus for participants. Walking emerged as a popular choice, with many incorporating it into their daily routines. Additionally, the practice of Surya Namaskar (a yoga sequence) was mentioned by several participants as part of their exercise regimen. The role of family and friends in maintaining motivation was highlighted, with some participants enjoying walks with loved ones.

"Yes, I walked daily for half an hour to an hour." - IDI-1

"I practice Surya Namaskar and walk for at least an hour daily." - IDI-2

Overcoming barriers with regular physical activity: Participants discussed overcoming physical and logistical challenges in maintaining regular physical activity. Some found yoga initially challenging due to time constraints or physical limitations. However, many adapted by finding alternative exercises or gradually incorporating physical activity into their routines. Despite facing challenges, participants expressed determination and a commitment to maintaining their exercise routines.

"I walk approximately three kilometers daily from home to work." - IDI-10

"Actually, I don't specifically go for walks as I work in the fields, which involves a lot of physical activity." - IDI-11

Theme 3: Support System and Motivation

The third theme, Support System and Motivation, delves into the crucial role of family, healthcare providers, and work-life balance in participants' weight loss journeys. Participants share insights into the supportive networks that propelled them forward, emphasizing the significance of interpersonal relationships, nurse-led guidance, and intrinsic motivation.

Family support and work-life balance: Family support was identified as a significant factor in participants' health journeys. Spouses and family members served as motivators, encouraging participants to prioritize their health. Participants managed to integrate lifestyle changes without disrupting family or work commitments, highlighting the importance of balance and communication within the family unit.

"Absolutely! My husband was my biggest motivator." - IDI-1

"Initially, it was challenging, but now they are supportive and pleased with my progress." - IDI-3

Reflection and realization: Participants reflected on the positive changes they experienced through their weight loss and lifestyle modifications. They acknowledged the importance of exercise and proper nutrition in achieving and maintaining a healthy lifestyle. Many shared their improved health conditions, increased happiness, and the financial benefits of adopting healthier habits.

Theme 4: Insights and Strategies from Participants

In the theme of Weight Maintenance and Guidance for Others, participants offered valuable advice and strategies for weight management following the intervention. They reflected on their experiences, sharing insights and recommendations for sustaining weight loss and assisting others on similar health paths.

Strategies employed for achieving success: Participants shared strategies they found effective for successful weight management and overall well-being. They emphasized the importance of adhering to diet charts, maintaining daily routines, prioritizing health, and being proactive in making healthy choices. These strategies were seen as essential for achieving and maintaining long-term health and wellness goals.

Advice for weight loss and overall well-being: Based on their experiences, participants offered advice to others seeking to improve their health. Recommendations included focusing on seasonal and balanced diets, incorporating regular exercise, and making small, sustainable changes to diet and lifestyle.

"Eat seasonal fruits like watermelon, jackfruit, and mangoes. Enjoy them in moderation and stay active throughout the day." - IDI-1

"I would advise them to focus on a balanced diet rich in vegetables and fruits, along with regular physical activity." - IDI 9

## Discussion

The demographic profile of the participants in this study provides valuable insights into the intersectionality of age, education, occupation, and cultural background in the context of weight management. Younger adults are prone to adopting unhealthy habits and gaining weight [12]. The substantial number of younger adults in this study actively seeking weight management interventions underscores their proactive approach to preventing future health issues. Adults with lower educational attainment often report poorer general health [13]. However, in this study, the significant representation of individuals with primary education who achieved weight loss success and were willing to share their experiences suggests their readiness to seek out and utilize health information and resources.

The observed improvements in clinical parameters such as body weight, BMI, and blood pressure corroborate the efficacy of NLIs in promoting positive health outcomes [14]. These findings resonate with studies demonstrating that structured weight management programs led by healthcare professionals can significantly impact metabolic health [15].

Participants highlighted the importance of including protein-rich foods and millets in their diets. Their focus on mindful eating and consuming seasonal fruits and vegetables aligns with dietary recommendations for Indians. Additionally, the dietary changes made by participants, such as reducing carbohydrate intake, choosing natural sweeteners, and limiting oil consumption and unhealthy snacks, are in line with Indian dietary guidelines [16].

The challenges faced by participants in adapting to new dietary habits, including managing hunger and navigating dietary changes within family dynamics, resonate with findings from studies highlighting the complexities of dietary behavior change and the importance of social support [17].

The preference for walking and yoga among participants aligns with global recommendations emphasizing the role of moderate-intensity physical activity in weight management and overall well-being [18]. The incorporation of physical activity into daily routines underscores the importance of sustainable exercise habits for long-term health [19].

The challenges faced by participants in maintaining regular physical activity, such as time constraints and physical limitations, are commonly reported barriers to exercise adherence [20]. The strategies adopted by participants to overcome these barriers highlight the importance of flexibility and adaptability in sustaining physical activity routines [21].

The role of family support in participants' weight loss journeys echoes findings from previous research highlighting the positive impact of social support networks on lifestyle modifications and adherence to health behaviors [22]. The emphasis on work-life balance underscores the importance of integrating health-promoting activities into daily routines without disrupting family or work commitments.

The participants' reflections on the positive changes experienced through weight loss and lifestyle modifications resonate with studies demonstrating the psychological benefits of improved health and well-being [23].

The advice and recommendations shared by participants for weight maintenance and overall well-being align with evidence-based guidelines advocating for balanced nutrition, regular physical activity, and sustainable lifestyle changes [24].

The strategies identified by participants for successful weight management, such as adhering to diet charts and maintaining daily routines, mirror recommendations for long-term weight loss maintenance and overall health [25].

Limitations of the study

While this study provides valuable insights into the experiences and strategies of adults who successfully lost weight through NLIs, there are several limitations to consider. The sample size of 13 participants, while sufficient for qualitative analysis, may not be representative of the broader population, limiting the generalizability of the findings. Additionally, the demographic profile of predominantly younger adults with primary education may not reflect the diversity of individuals seeking weight management interventions. The reliance on self-reported data for dietary and physical activity behaviors introduces potential recall and social desirability bias, which could affect the accuracy of the reported experiences [26]. Although the study followed participants for 12 months, the lack of further long-term follow-up data limits our understanding of the sustainability of the observed improvements in clinical and behavioral parameters. Future research with extended follow-up could offer valuable insights into the long-term effectiveness of NLIs. Lastly, while qualitative research offers rich insights, integrating these findings with quantitative data could provide a more comprehensive understanding of the factors influencing weight loss outcomes [27,28].

Summary of the study

This qualitative study explored the experiences of 13 adults who successfully lost at least 5% of their weight through NLIs. The study aimed to shed light on the strategies, challenges, and motivations that shaped their weight loss journeys. The participants, predominantly middle-aged, Hindu, and married individuals with primary education, reported positive health improvements over a 12-month period. These improvements included reductions in body weight, BMI, waist circumference, blood pressure, glucose levels, and lipid profiles.

Participants emphasized the adoption of protein-rich foods, millets, and a diverse range of fruits and vegetables in their diets. They also highlighted the importance of mindful eating and informed nutritional choices. Walking and Surya Namaskar (a yoga sequence) emerged as popular forms of physical activity among participants. Family and friends played a crucial role in maintaining motivation.

Family support, healthcare providers, and work-life balance were identified as significant factors in participants' weight loss journeys. Participants reflected on the positive changes they experienced and shared insights into the supportive networks that propelled them forward.

Participants offered valuable advice and strategies for weight management post-intervention. They emphasized the importance of seasonal and balanced diets, regular exercise, and making small, sustainable changes to diet and lifestyle.

## Conclusions

The findings from this study offer valuable insights into the experiences, challenges, and strategies adopted by adults who have successfully lost weight through NLIs. The observed improvements in clinical parameters and the adoption of healthier dietary and lifestyle habits underscore the effectiveness of NLIs in promoting sustainable weight loss and improved metabolic health. Despite its contributions, the study has several limitations, including a small sample size, reliance on self-reported data, lack of long-term follow-up, and potential confounding factors that were not addressed.

Future research addressing these limitations could further enhance our understanding of the complex factors influencing weight management success and inform the development of tailored interventions to meet the diverse needs of individuals seeking to improve their health through sustainable lifestyle changes.

## Appendices

Semi-structured in-depth interview guide for the adults who successfully lost the clinically significant weight loss of ≥ 5%

Introduction

Hello. Thank you for coming and participating in this interview. My name is Vembu and I am a PhD scholar from JIPMER, Puducherry. Working on my thesis, regarding obesity and weight loss. The conversation will take approximately 30-45 minutes. From this point on, the conversation will be audio-taped. The digital audio recordings will only be used to make transcripts of the interview, referring to you by your special identifier number and not by your name. Are there any questions or concerns before we begin?

Intro question: Today, I will just interview you as you underwent weight loss successfully. How much weight have you lost during the nurse-led intervention?

1.      Did you have any determination to reduce your weight before enrolling in the nurse-led wellness program?

2.      Can you talk about the overall experience of your weight loss journey?

3.      Have you set any goals for reducing weight?

Probe: Have you changed your eating behavior? If yes, what kind of diet you have taken? How do you consistently stick to your eating pattern?

4.      How much time do you spend on exercises and what type of exercise?

5.      Any physical activity modification did you adopt in your daily activities?

6.      What motivated you to lose weight?

7.      What are all the issues faced during weight loss?

Probe: How did you overcome those issues (one by one?)

8.      What is your advice for those who have not taken the steps to reduce weight?

Probe: on diet?

On exercise?

Any common issues?

I came to know all your aspects of the weight loss journey. These are all things that may be helpful for others who want to reduce weight. I hope you will set an example for others by maintaining this healthy weight loss.

Closing: Thank the participant for their time and for sharing their experiences.
